# Effects of *Trametes versicolor*-*Pseudostellaria heterophylla* Fermented Substrate on Growth Performance, Rumen Function and Meat Quality of Kazakh Lambs

**DOI:** 10.3390/ani16172792

**Published:** 2026-09-05

**Authors:** Changzhao Jin, Ayiguli Abudoukelimu, Zainafuguli Aishanjiang, Yong Chen, Jiancheng Liu

**Affiliations:** 1 Research Center for Biological Feed and Animal Gut Health, College of Animal Science, Xinjiang Agricultural University, Urumqi 830052, China; j17799683492@163.com (C.J.);; 2 Xinjiang Han Ziran Biotechnology Co., Ltd., Changji 831199, China

**Keywords:** *Trametes versicolor*, *Pseudostellaria heterophylla*, Kazakh lambs, growth performance, rumen function, meat quality

## Abstract

This experiment was conducted to investigate the effects of dietary supplementation with *Trametes versicolor*-*Pseudostellaria heterophylla* fermented substrate (TV-PHFS) on the growth performance, serum biochemical indices, rumen function, and meat quality of Kazakh ram lambs. Twenty-four Kazakh ram lambs (32.54 ± 5.23 kg) were randomly allocated into four groups, with six replicates per group and one lamb per replicate. The control group was fed a basal diet, while the experimental groups received the basal diet supplemented with 0.1, 0.2, and 0.3 kg/d of TV-PHFS, respectively, for a total duration of 140 days. The findings indicated that basal diet supplementation with TV-PHFS did not exert a statistically significant effect on the growth performance, serum antioxidant indices, and immune indices of Kazakh ram lambs (*p* > 0.05). However, compared with the control group, supplementation with 0.2 kg/d of TV-PHFS significantly elevated the 45 min postmortem yellowness (b*) value of the *longissimus dorsi* muscle and the relative abundance of *Prevotellaceae_UCG_003* in the rumen (*p* < 0.05). Similarly, supplementation with 0.3 kg/d of TV-PHFS significantly increased the 24 h postmortem lightness (L*) value and crude fat content of the *longissimus dorsi* muscle, as well as the relative abundances of *Treponema* and *Veillonellaceae_UCG_001* in the rumen (*p* < 0.05). In conclusion, basal diet supplementation with 0.3 kg/d of TV-PHFS modulated the rumen microbial community structure, promoted intramuscular fat deposition, improved muscle coloration, and augmented meat quality in Kazakh ram lambs.

## Simple Summary

*Pseudostellaria heterophylla* is a valuable traditional Chinese medicinal product used for health care, renowned for its rich composition of various bioactive constituents and its multiple physiological and pharmacological effects. By-products such as root fibrils and tail roots of *Pseudostellaria heterophylla* contain functional components resembling those of the main root and hold potential value as functional feed ingredients or feed additives. In the present study, the medicinal fungus *Trametes versicolor* was employed for the solid-state fermentation of *Pseudostellaria heterophylla* root fibrils to produce a *Trametes versicolor*-*Pseudostellaria heterophylla* fermented substrate (TV-PHFS), which was subsequently incorporated into the basal diet of Kazakh lambs during a feeding trial. The findings demonstrated that dietary supplementation with 0.3 kg/d of the TV-PHFS modulated rumen fiber-degrading microbiota, promoted intramuscular fat deposition, improved muscle coloration, and enhanced the quality of lamb meat in Kazakh lambs. This research provides a theoretical foundation for the high-value utilization of *Pseudostellaria heterophylla* root by-products and the development of innovative functional feed additives.

## 1. Introduction

In ruminant production, feed additives and alternative feed resources have received increasing attention, particularly with regard to the utilization of agricultural or processing by-products to develop high-value feeds, which has contributed to improving production efficiency and sustainability. As examples, Ahmadzadeh-Gavahan et al. [1] reported that the combined administration of propylene glycol and rumen-protected choline chloride in the ewes’ restricted diet could improve placental characteristics and subsequently improve lambs’ birth weight and body size. Boukrouh [2] reported that moderate inclusion of fresh or shade-dried Azolla in sheep diets improved body weight gain, feed conversion ratio, carcass traits, and economic efficiency. Fungal solid-state fermentation is an effective approach to improve the feeding value of lignocellulose-rich plant substrates, and its mechanism of action is mainly attributed to the extracellular enzymes secreted by fungi, which could modify the lignocellulosic structure and thereby improve the nutritional composition and physicochemical properties of the fermented products [3]. Feng et al. [4] reported that solid-state fermentation could improve the digestibility of plant-based proteins, decompose macromolecular proteins into bioactive small peptides, alter the amino acid profile, and eliminate anti-nutritional factors. Therefore, the present experiment was conducted with Kazakh lambs to investigate the effects of fermented Chinese herbal by-products included in their diet on the lambs’ growth performance, rumen fermentation function, and meat quality.

Fungal solid-state fermentation could produce various bioactive substances, including polysaccharides, enzymes, and secondary metabolites, which might exert regulatory effects on the rumen microbial community. Previous studies indicated that fungal cultures could serve as potential prebiotics, promote rumen development and the proliferation of fiber-degrading bacteria in sheep, thereby influencing rumen fermentation pathways and affecting the concentrations of propionate, butyrate, and total volatile fatty acids [5]. *Trametes versicolor* belongs to the phylum Basidiomycota, class Agaricomycetes, order Polyporales, and family Polyporaceae. It is a widely distributed macrofungus responsible for wood decay globally [6,7,8]. As a traditional medicinal fungus, *Trametes versicolor* is rich in bioactive substances such as polysaccharides, glycopeptides, terpenoids, and sterols [9,10,11]. Moreover, *Trametes versicolor* secretes extracellular enzymes such as laccase, ligninase, and cellulase, which effectively degrade macromolecular substances like lignin and cellulose into smaller, more easily absorbed nutrients [12,13]. These characteristics give it unique advantages in the field of feed resource development.

*Pseudostellaria heterophylla*, also known as Tong Shen, Mi Shen, or Si Ye Shen, is the tuberous root of the perennial herbaceous plant Haiershen of the family Caryophyllaceae, and was rich in various bioactive components such as polysaccharides, cyclic peptides, saponins, volatile oils, alkaloids, sterols, amino acids, fatty acids, and trace elements, serving as an important resource for the development of Chinese herbal medicines and health foods [14,15,16]. The root by-products of *Pseudostellaria heterophylla* contain a variety of amino acids that are essential for animals, with relatively high levels of functional amino acids such as arginine and γ-aminobutyric acid [17,18]. Furthermore, *Pseudostellaria heterophylla* contains various mineral elements, among which K, Ca, P, Fe, and Mg are the most abundant, followed by Zn, Mn, Cu and others [19]. *Pseudostellaria heterophylla* is mainly used as a medicinal material for human health care [14,20]; however, during its harvesting and processing, large quantities of by-products such as root fibrils and tail roots (accounting for approximately 10–15% of the total tuberous root yield) are often discarded [21]. The active component of these by-products is similar to that of commercial *Pseudostellaria heterophylla* roots, indicating potential value for development as functional feed ingredients or additives for livestock. However, studies on *Pseudostellaria heterophylla* and its by-products as feed additives have been conducted almost exclusively in monogastric animals such as pigs and poultry, while their effects in ruminants remain unreported. Although the bi-directional solid-state fermentation of *Pseudostellaria heterophylla* root by-products with *Trametes versicolor* has been described in previous work, it has not been applied in animal trials. To the best of our knowledge, this study is the first to incorporate TV-PHFS into ruminant diets and to evaluate its effects on the growth performance, rumen fermentation, and meat quality of Kazakh lambs.

Bidirectional fermentation is a process in which Chinese medicinal materials are added to traditional solid-state fermentation substrates as medicinal substrates, enabling bidirectional interactions between fungi and the medicinal materials. Compared with traditional solid-state fermentation, it possessed both the transformation effects of fungi on herbal components and the regulatory effects of the medicinal materials on fungal metabolism, with advantages such as enhancing efficacy, detoxification, and promoting the secondary development of Chinese medicinal resources [22]. Therefore, in the present study, the rootlets and tail roots of *Pseudostellaria heterophylla* were used as the medicinal substrate, and *Trametes versicolor*, which was rich in enzyme production and diverse in activities, was selected as the fermentation strain to prepare the “*Trametes versicolor*-*Pseudostellaria heterophylla* fermented substrate” through bidirectional fermentation technology. Previous studies indicated that TV-PHFS could undergo beneficial changes in nutritional composition and some biological activities [23]. However, its effects in ruminants have not been reported. Therefore, in the present study, TV-PHFS was added as a functional feed additive to the diet of Kazakh lambs to systematically investigate its effects on their growth performance, rumen function, and meat quality. This was expected to provide a theoretical basis and practical reference for the high-value utilization of *Pseudostellaria heterophylla* root by-products and the development of novel functional feed additives.

## 2. Materials and Methods

### 2.1. Ethical Statement

All animal experiments were conducted according to the relevant national guidelines and were approved by the Animal Welfare Care and Use Committee of Xinjiang Agricultural University (Xinjiang, China) (Animal protocol number: 2024006). Sampling operations were strictly conducted in accordance with the relevant provisions of the “Guiding Opinions on the Humane Treatment of Experimental Animals” issued by the Ministry of Science and Technology of the People’s Republic of China (Guo Ke Fa Cai Zi [2006] No. 398).

### 2.2. Test Materials

The *Trametes versicolor* strain GDMCC 5.178 was purchased from the Guangdong Microbial Culture Collection Center (GDMCC), Guangdong Institute of Microbiology. The strain is publicly available under the accession number GDMCC 5.178, and its basic identification information is provided by the culture collection. The root and tail root of *Pseudostellaria heterophylla* (hereafter referred to as *Pseudostellaria heterophylla*) were acquired from local farmers in Zherong County, Fujian Province, China.

The preparation of TV-PHFS followed the protocol described by Yang et al. [23], comprising 59% corn cob, 10% *Pseudostellaria heterophylla*, 29% bran, 1% gypsum, and 1% lime, with a moisture content of 62%. The medium was sterilised at 121 °C for 1.5 h, then cooled and inoculated with activated *Trametes versicolor* fungus solid seed at an inoculation rate of 2%. The inoculated mixture was incubated at 25 °C for 10 days. Following fermentation, the sample was dried at 50 °C to achieve a constant weight, pulverised through a 40-mesh sieve, and subjected to nutritional analysis. The analytical methods were as follows: gross energy was determined using an oxygen bomb calorimeter [24]; crude protein was determined by the Kjeldahl method with a nitrogen conversion factor of 6.25 [25]; crude fat was determined by Soxhlet extraction with petroleum ether [26]; crude ash was determined by incineration in a muffle furnace at 550 °C [27]; neutral detergent fiber was determined by the crucible method [28]; acid detergent fiber was determined by the filtration method [29]; calcium content was determined by EDTA disodium complexometric titration [30]; and phosphorus content was determined by spectrophotometry [31].

### 2.3. Experimental Design

Twenty-four healthy Kazakh male sheep, aged 4 months and weighing 32.54 ± 5.23 kg, were randomly selected and divided into four groups, with six sheep in each group, housed together in the same pen. The control group was fed daily with the basal diet, and the experimental groups received the basal diet supplemented with 0.1, 0.2, and 0.3 kg/day of TV-PHFS, respectively. The experiment lasted 140 days. The Kazakh lambs in each group were fed the same amount of basal diet daily. The composition and nutritional level of the basic diet are shown in Table 1.

### 2.4. Measurement of Body Weight

All experimental lambs were weighed after a 12 h fasting period on the initial day and the final day of the trial. The average daily gain (ADG) was determined using the initial and final weights, employing the formula.

(1) $$ADG\left(g/d\right)=\frac{Final\;weight\left(g\right)-Initial\;weight\left(g\right)}{Number\;of\;trial\;days(d)}$$

### 2.5. Determination of Biochemical Indicators in Serum

On the final day of the experiment, 10 mL blood samples were collected from each lamb from the jugular vein at 8:00 am. The samples were stored at 4 °C in a refrigerator for 30 min, then subjected to centrifugation at 3500 rpm using a low-speed centrifuge for 15 min to isolate the serum. According to the method of Niu et al. [32] the serum concentrations of total protein (TP), albumin (ALB), globulin (GLB), blood urea nitrogen (BUN), alanine aminotransferase (ALT), aspartate aminotransferase (AST), alkaline phosphatase (ALP), triglycerides (TG), total cholesterol (TC), high-density lipoprotein cholesterol (HDL-C), low-density lipoprotein cholesterol (LDL-C), and glucose (GLU) were quantified utilizing an automated blood biochemistry analyzer (TBA-FX8, Canon Medical Systems Corporation, Tochigi, Otawara-shi, Japan). Additionally, the ratios of albumin to globulin (ALB/GLB) and alanine aminotransferase to aspartate aminotransferase (ALT/AST) were calculated.

### 2.6. Determination of Antioxidant Indicators in Serum

Serum antioxidant indices, including superoxide dismutase (SOD) (A001-2-2, hydroxylamine method), glutathione peroxidase (GSH-Px) (A005-1-2, colorimetric method), catalase (CAT) (A007-1-1, ammonium molybdate method), malondialdehyde (MDA) (A003-1-2, TBA method), and total antioxidant capacity (T-AOC) (A015-2-1, ABTS method), were measured utilizing commercial assay kits supplied by Nanjing Jiancheng Bioengineering Institute, Nanjing, China.

### 2.7. Determination of Immune Markers in Serum

Serum immune indices, including immunoglobulin A (IgA) (ml025601), immunoglobulin M (IgM) (ml025607), immunoglobulin G (IgG) (ml025602), interleukin-1β (IL-1β) (ml301074), tumor necrosis factor-α (TNF-α) (ml301072), and interleukin-10 (IL-10) (ml824689), were measured using commercial ELISA kits obtained from Shanghai Enzyme-linked Biotechnology Co., Ltd., Shanghai, China.

### 2.8. Measurement of Rumen Fermentation Parameters

At the end of the experiment, all lambs were humanely euthanized by exsanguination after stunning. Subsequently, the abdominal cavity was promptly opened to facilitate the collection of rumen contents. These contents were filtered through four layers of gauze, and the rumen fluid was transferred into sterile cryovials, rapidly frozen in liquid nitrogen, and stored at −80 °C. The pH of the rumen fluid was measured using a Shanghai Leimu pH meter (Shanghai Yidian Scientific Instrument Co., Ltd., Shanghai, China). Volatile fatty acids (VFAs) concentrations were determined according to the method of Wei et al. [33] using a Shimadzu gas chromatograph (GC-2010, Shimadzu, Kyoto, Japan) with 4-methylvaleric acid as the internal standard, and the total VFA (TVFA) content as well as the acetate-to-propionate ratio were calculated. The NH_3_-N concentration was determined by the phenol–sodium hypochlorite colorimetric method [34].

### 2.9. Determination of Rumen Microbial 16S rDNA

DNA extraction and PCR amplification of rumen fluid samples were conducted by Novogene. The V3-V4 hypervariable region of the 16S rDNA gene was amplified utilizing primers 341F (5′-CCTAYGGGRBGCASCAG-3′) and 806R (5′-GGACTACNNGGGTATCTAAT-3′). The PCR reaction mixture comprised 15 µL of Phusion High-Fidelity PCR Master Mix (New England Biolabs, Beijing, China), 0.2 µM of the reverse primer, and 10 ng of genomic DNA template. The resulting sequences, designated as Effective Tags, were subjected to denoising through the DADA2 module within QIIME2 (version 2022.2) to generate Amplicon Sequence Variants (ASVs) and feature tables. Species annotation was performed using QIIME2, and α diversity indices-including Observed_otus, Shannon, Simpson, and Chao1-were calculated. β diversity analysis was carried out based on weighted UniFrac distances. Venn diagrams were produced employing the Venn Diagram function in R software (version 4.0.3). The 10 most abundant species at the phylum and genus levels were visualized with bar plots generated utilizing the vegan and ggplot2 (version 4.0.3) packages. Differential species within the microbial community were identified through LEfSe analysis.

### 2.10. Assessment of Meat Quality

Meat quality was determined according to the method described by de Moraes Pinto et al. [35]. The *longissimus dorsi* on the left side of the back was selected, and pH measurements were recorded at 45 min and 24 h post-slaughter using a meat pH meter (HI981036, HANNA Instruments, Năsăud, Romania). Lightness (L*), redness (a*), and yellowness (b*) values were recorded at 45 min and 24 h post-slaughter utilizing a handheld colorimeter (CR-10, Konica Minolta, Tokyo, Japan). Shear force was tested by sampling a 2.0 cm × 3.5 cm × 5.0 cm section of the *longissimus dorsi* from the back within 1 h post-slaughter, sealing the sample in a plastic bag, and cooking it in an 80 °C water bath (SSW, Shanghai Boxun, Shanghai, China) for 10 min. The shear force was then measured using a muscle tenderness shear force tester (C-LM3B, College of Engineering, Northeast Agricultural University, Beijing, China). The remaining meat samples were stored at −20 °C for subsequent analysis of nutritional components and volatile flavor compounds. The moisture content of the muscle was determined using an electric heating constant temperature blast drying oven (DHG-9145 A, Shanghai Qixin, Shanghai, China). The method was based on GB 5009.3-2016 [36]. From each lamb, a 10 g muscle sample was collected from the *longissimus dorsi* and placed in a 105 °C constant-temperature drying oven until a stable weight was achieved. The moisture content was calculated based on the weight difference before and after drying. Ash content was measured using a muffle furnace (SX-4-10T, Beijing Guangming, Beijing, China). The method was based on GB 5009.4-2016 [37]. Samples were initially calcined at 200 °C for 1.5 h, followed by calcination at 500 °C for 2.5 h. The ash content was computed from the residual ash after combustion. Crude protein content was assessed via the Kjeldahl method. The method was based on GB 5009.5-2025 [38]. Following digestion, nitrogen content was measured with an automatic Kjeldahl nitrogen analyzer (K1100, Hanon, Jinan, China), and crude protein was determined by multiplying the nitrogen content by a conversion factor. Lipid content was determined through Soxhlet extraction with petroleum ether as the solvent. The method was based on GB 5009.6-2025 [39] utilizing an automatic fat analyzer (Sox406, Hanon, Jinan, China) for reflux extraction. The fat content was ascertained by calculating the difference in mass of the sample prior to and following the extraction process. Backfat thickness was measured by sectioning the carcass at the end of the 12 th rib and employing a digital vernier caliper (JZYQ-001, Ningbo Mingyan Tools Co., Ltd., Ningbo, China) to measure the subcutaneous fat thickness 1 cm from the rib’s end along the dorsal midline. 

### 2.11. Determination of Volatile Flavor Compounds in Lamb Meat

Volatile flavor compounds in the muscle samples were determined according to the method described by Ge et al. [40]. An exact weight of 5.0 g of the *longissimus dorsi* muscle sample was precisely measured. Under conditions maintained with an ice bath, the sample was homogenized utilizing a tissue grinder and subsequently transferred into a 40 mL colorless, odorless, transparent headspace sampling vial. The vial was then sealed with sealing film and covered with a cap. It was allowed to equilibrate at ambient temperature for a duration of 30 min. Measurement was carried out using an electronic nose (cNose-14, Shanghai Baosheng, Shanghai, China). The sensor probe was inserted approximately halfway into the headspace vial, employing air as the carrier gas. The sampling duration was set to 120 s with a flow rate of 1 L/min to analyze the volatile flavor compounds of raw meat. Following each sample measurement, the sensors were cleansed for 120 s until the sensor curves stabilized, in order to prevent measurement interference. After the raw meat assessment, the same headspace vial was placed in a water bath set at 80 °C for 10 min, then removed and allowed to cool naturally to room temperature. The volatile flavor compounds of cooked meat were subsequently measured using the same procedural approach described above.

### 2.12. Statistical Analyses

Experimental data were organized using Excel 2021 and analyzed using SPSS 27.0 (IBM), with statistical significance set at *p* < 0.05. Initial body weight was analyzed by one-way analysis of variance (ANOVA), and homogeneity of variance was assessed by Levene’s test; if variances were homogeneous, pairwise comparisons between groups were performed using the LSD method and Duncan’s multiple range test. Average daily gain and final body weight were analyzed by analysis of covariance (ANCOVA) with group as the fixed factor and initial body weight as the covariate. The group × initial body weight interaction was first tested; if the group × initial body weight interaction was not significant (*p* > 0.05), satisfying the homogeneity of slopes assumption, the interaction term was removed and the main effect of group was reanalyzed. Post hoc multiple comparisons were performed using the Bonferroni method to compare estimated marginal means, and polynomial contrasts were used to test linear and quadratic trends across levels, with the corresponding *p* values reported. Carcass weight, serum biochemical parameters, serum antioxidant and immune indices, rumen fermentation parameters, and meat quality traits were analyzed by one-way ANOVA with treatment group as the fixed factor, and homogeneity of variance was assessed by Levene’s test. If the ANOVA was significant, Duncan’s multiple range test was used for multiple comparisons; simultaneously, polynomial contrasts were used to analyze linear and quadratic trends of treatment effects, and the trend *p* values were reported.

## 3. Results

### 3.1. Nutritional Components and Bioactive Component Contents of TV-PHFS

TV-PHFS had an energy content of 16.90 MJ/kg, crude protein of 16.55%, crude fat of 2.40%, crude ash of 11.41%, neutral detergent fibre of 34.20%, acid detergent fibre of 22.36%, calcium of 0.41%, and phosphorus of 0.44%. The active components in TV-PHFS were as follows: glycopeptide content, 21.90 mg/g; polysaccharides, 10.03 mg/g; total phenolics, 6.87 mg/g; flavonoids, 1.93 mg/g; and triterpenoids, 1.57 mg/g [23].

### 3.2. Effects of TV-PHFS on Weight Fluctuations in Kazakh Lambs

Compared with the control group, supplementation with TV-PHFS did not significantly affect the final body weight or average daily gain of Kazakh lambs (*p* > 0.05); however, carcass weight showed a linear decreasing relationship (*p* = 0.044) (Table 2).

### 3.3. Effects of TV-PHFS on Biochemical Indicators in Kazakh Lamb Blood

Dietary supplementation with TV-PHFS did not significantly affect serum AST activity or TC content in Kazakh lambs (*p* > 0.05); however, with increasing TV-PHFS supplementation level, serum AST activity showed a quadratic response, decreasing first and then increasing (*p* = 0.032), whereas serum TC content showed a quadratic response, increasing first and then decreasing (*p* = 0.047). Supplementation with TV-PHFS had no significant effects on serum levels of TP, ALB, GLB, ALT, ALP, BUN, GLU, TG, HDL-C, and LDL-C in Kazakh lambs (*p* > 0.05) (Table 3).

### 3.4. Effects of TV-PHFS on Antioxidant Indicators in Kazakh Lamb Blood

Dietary supplementation with TV-PHFS did not significantly affect blood SOD, CAT, and GSH-Px activities, or MDA and T-AOC levels in Kazakh lambs (*p* > 0.05) (Table 4).

### 3.5. Effects of TV-PHFS on Immune Indicators in Kazakh Lamb Blood

Dietary supplementation with TV-PHFS did not significantly affect the concentrations of IgG, IgM, IgA, IL-1β, TNF-α, and IL-10 in the blood of Kazakh lambs compared with the control group (*p* > 0.05) (Table 5).

### 3.6. Changes in Rumen Fermentation in Kazakh Lambs Fed Different Quantities of TV-PHFS

Dietary supplementation with TV-PHFS did not significantly affect the butyrate concentration in the rumen fluid of Kazakh lambs (*p* > 0.05); however, with increasing TV-PHFS supplementation level, butyrate concentration showed a quadratic response, increasing first and then decreasing (*p* = 0.046). Supplementation with TV-PHFS had no significant effects on rumen fluid pH, the concentrations of acetate, propionate, isobutyrate, isovalerate, valerate, total volatile fatty acids, and ammonia nitrogen, or the acetate-to-propionate ratio in Kazakh lambs (*p* > 0.05) (Table 6).

### 3.7. Changes in Rumen Microorganisms in Kazakh Lambs Fed Different Quantities of TV-PHFS

**Figure 1:**
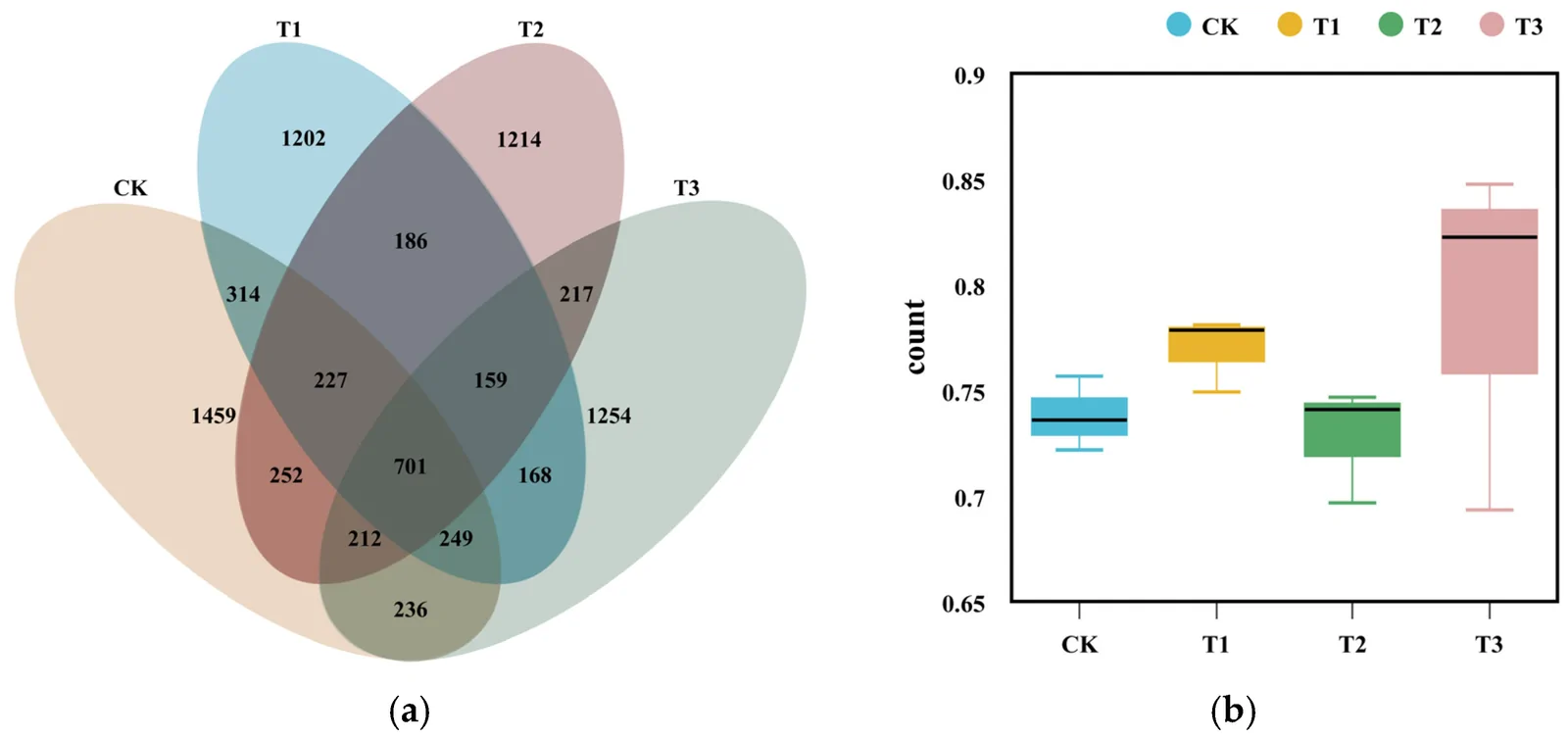
Venn diagram and β diversity index of the rumen microbial community in Kazakh lambs. (**a**): Number of rumen microbial ASVs; (**b**): β diversity index. CK: Control group (basal diet without TV-PHFS supplementation); T1: group supplemented with 0.1 kg/d of TV-PHFS; T2: group supplemented with 0.2 kg/d of TV-PHFS; T3: group supplemented with 0.3 kg/d of TV-PHFS.

Microbial ASV analysis across each group identified a total of 8050 ASVs within the four groups. Specifically, 701 ASVs were shared among all groups; 1459 ASVs were exclusive to the control group; 1202 ASVs were unique to the 0.1 kg/d group; 1214 ASVs were distinctive to the 0.2 kg/d group; and 1254 ASVs were specific to the 0.3 kg/d group. Furthermore, comparison with the control group revealed that the inclusion of TV-PHFS in the diet did not exert a statistically significant influence on the β diversity of rumen microbiota in Kazakh lambs (*p* > 0.05) (Figure 1).

**Figure 2:**
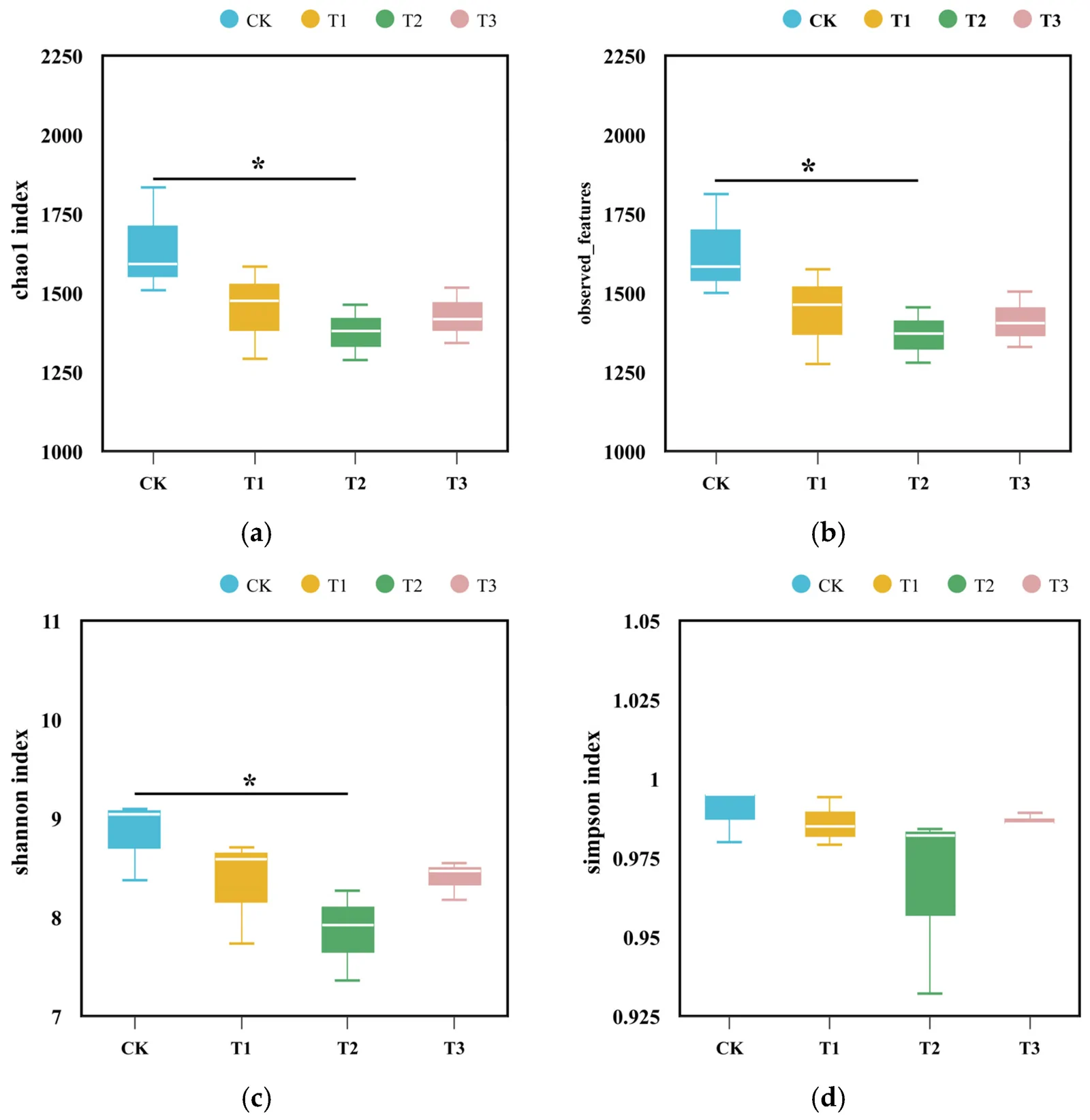
Effects of dietary TV-PHFS supplementation on rumen microbial alpha diversity of Kazakh lambs. (**a**): Chao1 index; (**b**): observed features index; (**c**): Shannon index; (**d**): Simpson index. *: *p* < 0.05. CK: control group (basal diet without TV-PHFS supplementation); T1: group supplemented with 0.1 kg/d of TV-PHFS; T2: group supplemented with 0.2 kg/d of TV-PHFS; T3: group supplemented with 0.3 kg/d of TV-PHFS.

The group of Kazakh lambs receiving 0.2 kg/d exhibited significantly lower alpha diversity indices (Chao1 index, Observed features index, and Shannon index) of rumen microorganisms in comparison to the control group (*p* < 0.05). No significant differences were observed between the 0.1 kg/d and 0.3 kg/d groups and the control group (*p* > 0.05). Additionally, the Simpson index of rumen microorganisms demonstrated no significant differences among all experimental groups and the control group (*p* > 0.05) (Figure 2).

**Figure 3:**
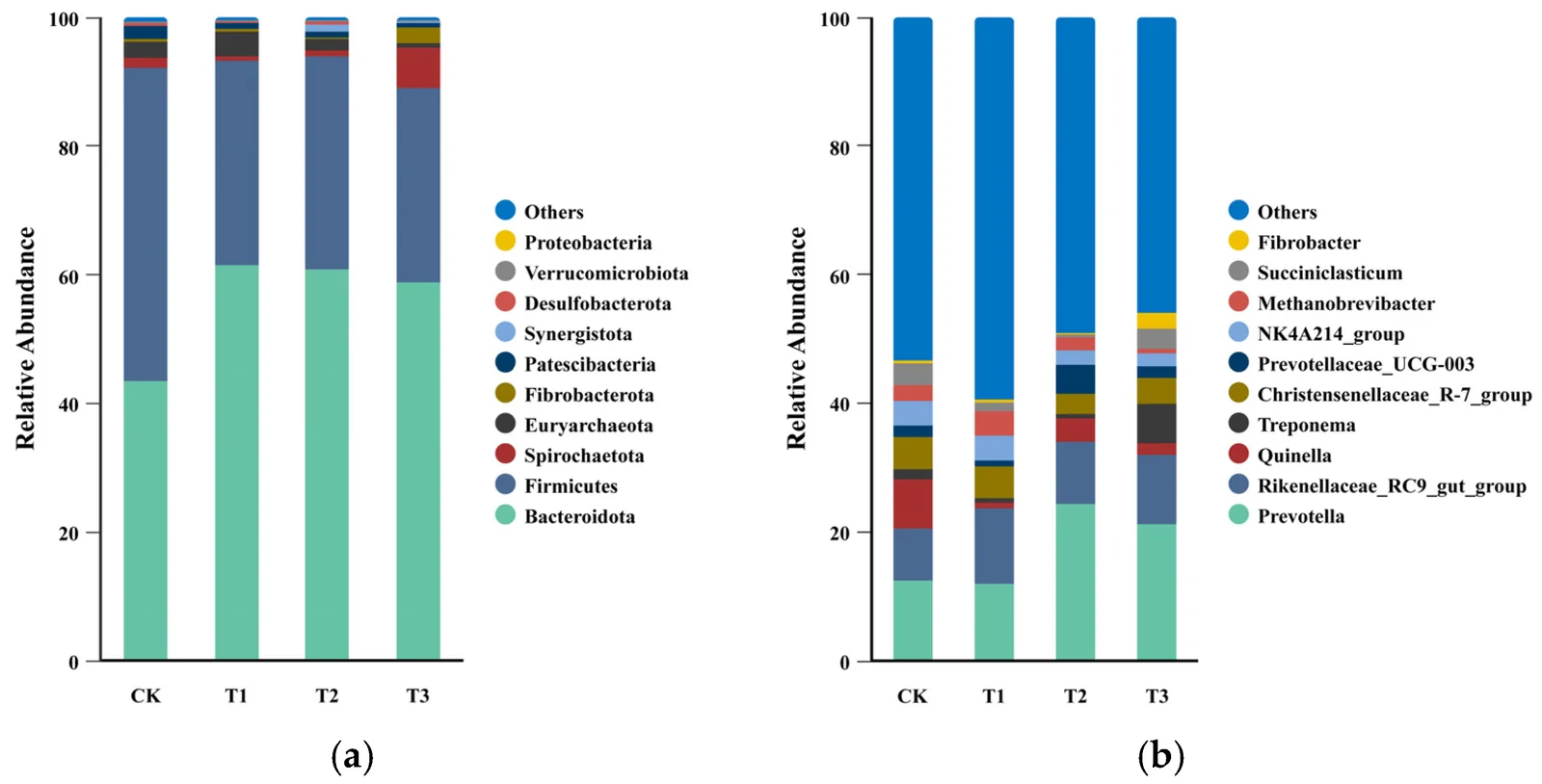
Relative abundance of the top 10 rumen microorganisms (**a**) at the phylum level; (**b**) at the genus level. CK: control group (basal diet without TV-PHFS supplementation); T1: group supplemented with 0.1 kg/d of TV-PHFS; T2: group supplemented with 0.2 kg/d of TV-PHFS; T3: group supplemented with 0.3 kg/d of TV-PHFS.

An analysis was conducted on the top 10 most relatively abundant phyla and genera within the rumen microbial community. At the phylum level, the experimental group with added TV-PHFS exhibited an increase in the relative abundance of *Bacteroidota*, whereas the relative abundance of *Firmicutes* declined compared to the control group. The 0.3 kg/d group demonstrated higher relative abundances of *Spirochaetota* and *Fibrobacterota* than the other three groups. At the genus level, the 0.2 kg/d and 0.3 kg/d groups displayed higher relative abundances of *Prevotella* than both the control group and the 0.1 kg/d group. The relative abundance of the *Rikenellaceae_RC9_gut_group* was greater in the treatment groups compared to the control group, while the control group exhibited a higher relative abundance of *Quinella* relative to the treatment groups (Figure 3).

**Figure 4:**
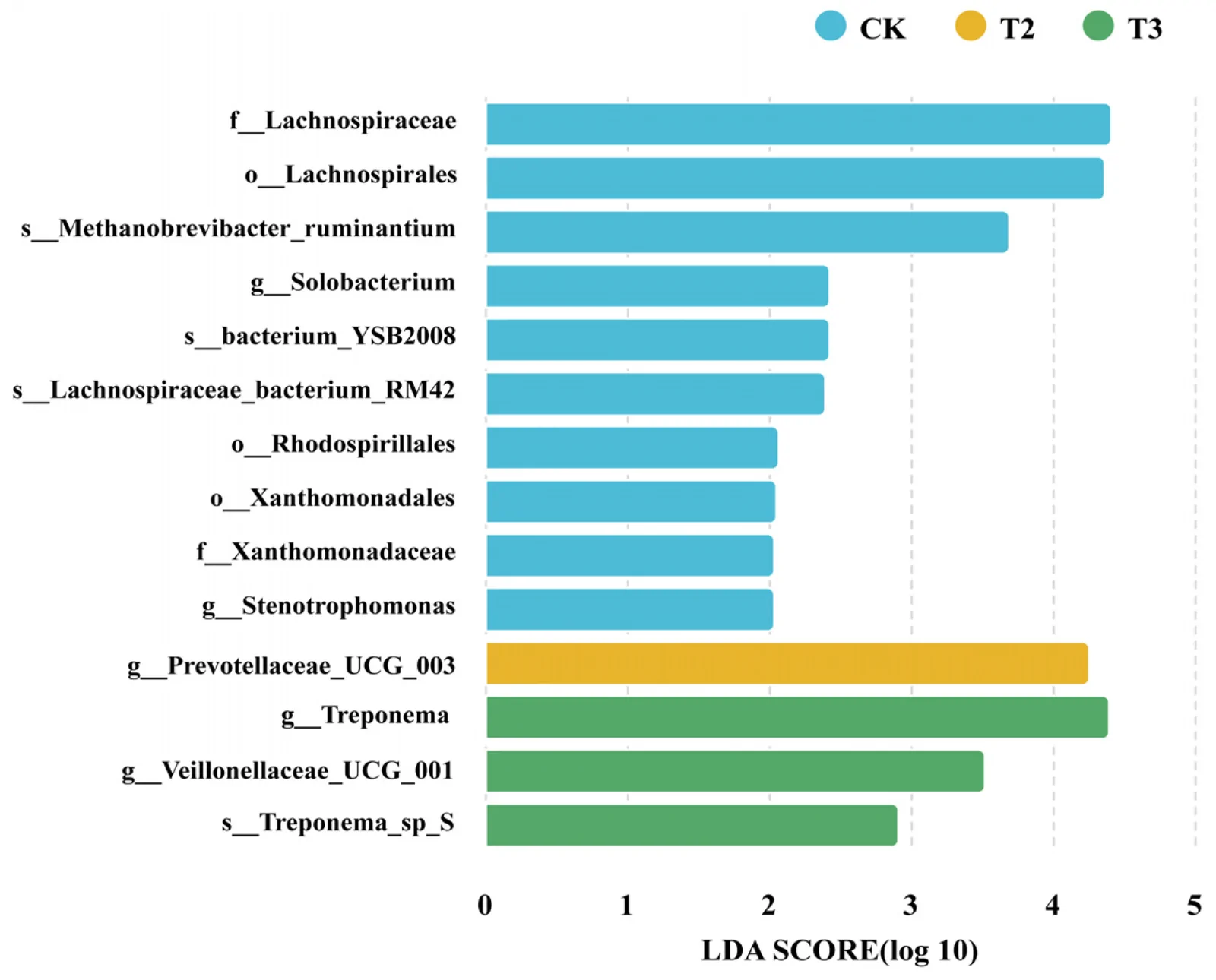
LEfSe analysis of the rumen microbiota in Kazakh lambs. CK: control group (basal diet without TV-PHFS supplementation); T2: group supplemented with 0.2 kg/d of TV-PHFS; T3: group supplemented with 0.3 kg/d of TV-PHFS.

When LDA > 2.0, LEfSe analysis was employed to investigate the effects of distinct bacterial communities, as illustrated in Figure 4. The findings identified 14 different bacterial taxa across various classification levels among the four groups, including 10 in the control group, one in the 0.2 kg/d group, and three in the 0.3 kg/d group. At the genus level, the relative abundances of *Solobacterium* and *Stenotrophomonas* were notably increased in the control group; *Prevotellaceae_UCG_003* exhibited a significant rise in the 0.2 kg/d group; and *Treponema*, along with *Veillonellaceae_UCG_001*, demonstrated significant increases in the 0.3 kg/d group.

### 3.8. Effects of TV-PHFS on the Meat Quality of Kazakh Lambs

Dietary supplementation with 0.3 kg/d TV-PHFS significantly increased the L* of the *longissimus dorsi* muscle of Kazakh lambs at 24 h postmortem compared with the control group (*p* < 0.05); dietary supplementation with 0.2 kg/d TV-PHFS significantly increased the b* of the *longissimus dorsi* muscle of Kazakh lambs at 45 min postmortem compared with the control group (*p* < 0.05). After TV-PHFS supplementation, the overall difference among groups in backfat thickness of Kazakh lambs was not significant (*p* > 0.05), but with increasing TV-PHFS supplementation level, the backfat thickness of Kazakh lambs showed a quadratic response, increasing first and then decreasing (*p* = 0.028). However, TV-PHFS supplementation had no significant effects on pH, a*, or shear force of the *longissimus dorsi* muscle of Kazakh lambs (*p* > 0.05) (Table 7).

### 3.9. Effects of TV-PHFS on the Nutritional Constituents of Kazakh Lambs Longissimus Dorsi Muscle

Compared with the control group, the addition of TV-PHFS in the diet had no significant effect on the moisture, crude ash, and crude protein content in the *longissimus dorsi* muscle of Kazakh lambs (*p* > 0.05). However, the addition of 0.3 kg/d of TV-PHFS significantly increased the crude fat content in the *longissimus dorsi* muscle of Kazakh lambs (*p* < 0.05) (Table 8).

### 3.10. Effects of TV-PHFS on Volatile Flavour Compounds in the Muscle of Kazakh Lambs

**Figure 5:**
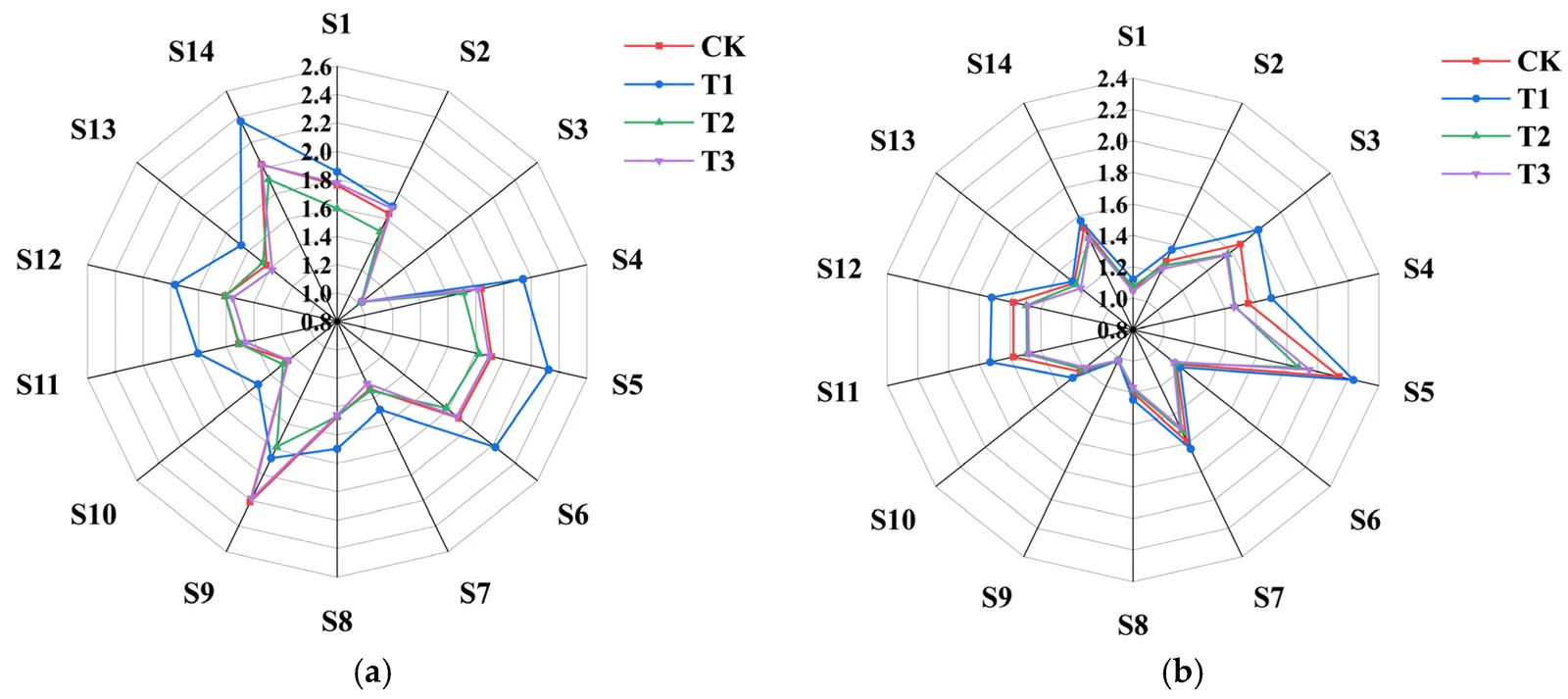
Effects of TV-PHFS on the volatile flavor compounds within the *longissimus dorsi* muscle of Kazakh lambs. The figure distinguishes between raw meat (**a**) and cooked meat (**b**), CK: control group (basal diet without TV-PHFS supplementation); T1: group supplemented with 0.1 kg/d of TV-PHFS; T2: group supplemented with 0.2 kg/d of TV-PHFS; T3: group supplemented with 0.3 kg/d of TV-PHFS.

The names of electronic nose sensors and their corresponding representative sensitive substances are presented in Table 9. The detection results indicated that the addition of 0.1 kg/d of TV-PHFS significantly increased the hydrogen (S10) compounds in raw Kazakh lamb meat (*p* < 0.05). A tendency towards an increase in alkane compounds (S8 and S13) was observed (*p* = 0.054, 0.090) (as illustrated in Figure 5a); concurrently, the cooked meat exhibited a tendency for an increase in propane/smoke-like compounds (S1) (*p* = 0.072). However, the addition of 0.2 and 0.3 kg/d of TV-PHFS did not exert a statistically significant effect on the volatile flavour compounds in the lambs (*p* > 0.05) (as illustrated in Figure 5).

## 4. Discussion

The present study demonstrated that dietary supplementation with TV-PHFS in the diet of Kazakh lambs did not significantly affect growth performance, which might be associated with several factors. During the preparation of TV-PHFS, approximately 88% carriers (59% corncob and 29% wheat bran) were added, whereas the *Pseudostellaria heterophylla* substrate accounted for only 10%, resulting in a relatively low amount of active components of *Pseudostellaria heterophylla* in the fermented product, which might not have reached the dose threshold required to exert growth-promoting effects. Currently, most studies on *Pseudostellaria heterophylla* are conducted in monogastric animals or in vitro; however, in ruminants, rumen microorganisms could degrade or transform plant-derived bioactive components, making it difficult for them to reach the hindgut in intact active forms to exert their effects [41]. In addition, the rumen microbiota and fermentation function of the lambs in the present experiment were already relatively mature, and the basal diet could basically meet their normal growth requirements; therefore, additional functional supplementation might struggle to exhibit significant growth-promoting effects.

Serum biochemical indices can objectively reflect nutrient digestion and metabolism, organ function, and health status in animals [42]. In the present study, serum AST activity in the 0.1 kg/d TV-PHFS group of Kazakh lambs tended to decrease (*p* = 0.059). Since AST is a marker of liver injury [43], this numerical trend might indicate a potential protective effect of TV-PHFS on hepatocyte integrity; however, this effect did not reach statistical significance, and no definitive conclusion could be drawn. Furthermore, other serum biochemical indices, including TP, ALB, GLB, ALT, ALP, BUN, GLU, TG, HDL-C, and LDL-C, were not significantly affected by TV-PHFS supplementation. This might be collectively attributed to several factors, such as an insufficient effective dose of active components, reduced bioavailability due to rumen microbial degradation, or the stable physiological state of the lambs.

Antioxidant activity, recognized as a crucial defense mechanism within organisms, is instrumental in maintaining redox homeostasis and preventing chronic diseases. Research has demonstrated that various bioactive compounds present in *Pseudostellaria heterophylla* exert antioxidant effects [14]. *Pseudostellaria heterophylla* polysaccharides could protect PC12 cells from H_2_O_2_-induced oxidative damage [44]. Additionally, polysaccharides and saponins derived from *Pseudostellaria heterophylla* significantly decrease MDA content, lower intracellular reactive oxygen species (ROS) levels, and increase SOD activity in CoCl_2_-induced H9c2 cardiomyocytes, thereby mitigating oxidative stress and safeguarding cells from oxidative injury [45]. Cyclic peptides from *Pseudostellaria heterophylla* have demonstrated the capacity to counteract oxidative stress and alleviate myocardial hypoxia-reoxygenation injury by inhibiting ROS production and enhancing mitochondrial function, thus exhibiting notable cardioprotective properties [46]. In the present study, compared with the control group, serum SOD activity in Kazakh lambs supplemented with 0.2 kg/d TV-PHFS was numerically higher, and MDA content was numerically lower, but the differences did not reach statistical significance (*p* > 0.05). Therefore, these results only suggested a possible trend of improvement in antioxidant capacity, and it could not be definitively concluded that TV-PHFS had significant antioxidant effects. This may be attributed to the active substances, such as polysaccharides present in TV-PHFS, being utilized and transformed within the rumen, thereby maintaining the redox state of the experimental subjects in homeostasis. Consequently, the antioxidant components in TV-PHFS primarily contributed to stability maintenance without producing a discernible enhancing effect.

Immune function serves as the fundamental defense mechanism of the body, responsible for recognizing and eliminating foreign substances and maintaining internal environmental homeostasis. Studies showed that the active components in *Pseudostellaria heterophylla*, such as cyclic peptides and polysaccharides, possessed immunomodulatory effects [14]. In vitro studies showed that active peptides isolated from *Pseudostellaria heterophylla* protein hydrolysates could promote the proliferation of mouse spleen lymphocytes and macrophage phagocytosis, and activate RAW264.7 cells to secrete NO, ROS, and TNF-α [47,48]. In animal models, *Pseudostellaria heterophylla* polysaccharides could enhance cellular and humoral immune functions in cyclophosphamide-induced immunosuppressed mice [49], and *Pseudostellaria heterophylla* decoction and its polysaccharides could increase immune organ indices and reduce pro-inflammatory cytokine levels in spleen-deficiency rats [50]. In addition, polysaccharides from *Trametes versicolor* were also reported to induce NO production, increase iNOS and TNF-α expression, and promote phagocytosis in RAW264.7 cells [51]. Studies in chicks further indicated that *Pseudostellaria heterophylla* polysaccharides could increase serum IgA, IgG, and IgM, increase CD3^+^ T lymphocyte subsets, enhance IFN-γ, IL-2, and lysozyme activities, and exert positive effects on spleen mRNA expression [52]. The present study found that dietary supplementation with TV-PHFS did not significantly affect serum levels of immunoglobulins and cytokines in Kazakh lambs (*p* > 0.05). This might be because the present experiment was conducted in ruminants, and the polysaccharides and other active components in TV-PHFS might be degraded or transformed by rumen microorganisms and could not reach the hindgut in intact active forms to exert immunomodulatory effects; on the other hand, the composition and dosage of active components in TV-PHFS differed from the extracted and purified products used in previous studies. Moreover, the present study only measured serum immune indices, so the potential effects of TV-PHFS on immune function in ruminants could not be completely ruled out.

Rumen fermentation parameters serve as vital indicators for evaluating the function of the rumen in ruminants, playing a significant role in regulating fermentation patterns and enhancing energy utilization efficiency. VFAs within the rumen constitute the primary energy source for ruminants [53]. Research has demonstrated that polysaccharides derived from *Artemisia ordosica* can promote the proliferation of fiber-degrading bacteria, thereby facilitating carbohydrate fermentation and increasing VFAs production [54]. Xiao et al. [50] showed that polysaccharides from *Pseudostellaria heterophylla* could markedly elevate butyrate levels in the cecum of rats with spleen-deficiency syndrome, indicating their potential to stimulate gut microbiota to produce higher quantities of butyrate. Butyrate is known to promote the proliferation and differentiation of rumen epithelial cells [55], enhance VFAs absorption [56], and improve ruminal fermentation function. The present investigation revealed that dietary supplementation with the TV-PHFS did not significantly influence the concentrations of acetate, propionate, isobutyrate, isovalerate, and total VFAs in the rumen of Kazakh lambs (*p* > 0.05). This lack of significant effect may be attributed to the maturation of the rumen in the experimental lambs and the attainment of a steady internal environment, as well as the possibility that the active component dosage in TV-PHFS did not reach the threshold necessary to modify fermentation patterns. The ruminal NH_3_-N concentrations in all TV-PHFS-supplemented groups of Kazakh lambs were numerically higher than that in the control group, but the difference did not reach statistical significance (*p* = 0.133). This observation could be explained by the higher crude protein content in TV-PHFS, which, to some extent, increased the dietary crude protein level, thereby elevating NH_3_-N concentrations. Additionally, the trypsin inhibitors present in *Pseudostellaria heterophylla* [57] may have reduced the utilization of crude protein by rumen microorganisms; however, the precise mechanisms warrant further investigation.

The rumen of ruminants constitutes a complex microecosystem, with the rumen microbiota playing a crucial role in nutrient absorption and utilization [58]. Research has demonstrated that bioactive compounds in *Pseudostellaria heterophylla* can enhance intestinal microecology and promote host health by modulating the gut microbiota and its metabolites. *Pseudostellaria heterophylla* polysaccharides have been shown to decrease the relative abundance of *Firmicutes* and increase that of *Bacteroidetes* in the cecum of rats with spleen-deficiency syndrome, significantly elevating the levels of *Lactobacillus* and *Alloprevotella* while decreasing *Romboutsia* [50]. Furthermore, Chen et al. [59] reported that cyclic peptides derived from *Pseudostellaria heterophylla* could rectify gut microbiota dysbiosis in mice with dextran sulfate sodium (DSS)-induced colitis, elevating the abundance of beneficial bacteria such as *Firmicutes*, *Bacteroidetes*, *Bilophila*, *Micromonospora*, and *Dubosiella*, thereby enhancing intestinal barrier integrity and preventing or alleviating colonic inflammation. In the present study, dietary supplementation with 0.2 kg/d of TV-PHFS significantly decreased the Chao1 index, observed species, and Shannon index of the rumen microbiota. Concurrently, a distinct directional shift in microbial community composition was observed, characterized by an increased relative abundance of *Bacteroidota* and a significant enrichment of fiber-degrading bacterial genera such as *Prevotella* and *Prevotellaceae_UCG-003*. Accordingly, the reduction in α-diversity was not indicative of stochastic species loss but rather reflected a selective promotion of fiber-degrading bacterial populations. Notably, this community restructuring occurred without significant changes in the Simpson index or β-diversity, suggesting that the stability of the dominant microbial community and the overall community structure were largely maintained, while the functional capacity for fiber degradation may have been enhanced. Compared to the control group, all three treatment cohorts exhibited an increased relative abundance of *Bacteroidota* and a decreased presence of *Firmicutes*. *Bacteroidota* primarily generate energy by degrading carbohydrates and proteins, whereas *Firmicutes* are mainly involved in volatile fatty acid production through cellulose degradation [60]. The shift in their relative abundances may be attributable to the higher amounts of polysaccharides, crude proteins, and crude fats in TV-PHFS, which facilitate the proliferation of *Bacteroidota*. At the genus level, the relative abundance of *Prevotella* in the 0.2 and 0.3 kg/d groups was greater than that observed in the control and 0.1 kg/d groups. *Prevotella* is one of the dominant bacterial genera in the rumen, primarily involved in hemicellulose degradation [61], implying that supplementation with medium and high concentrations of TV-PHFS might specifically enrich fiber-degrading bacterial populations, thereby enhancing crude fiber digestibility. LEfSe analysis revealed a significant increase in the relative abundance of *Prevotellaceae_UCG-003* in the 0.2 kg/d group, while *Treponema* and *Veillonellaceae_UCG-001* were notably elevated in the 0.3 kg/d group. These microorganisms are known to facilitate the degradation of hemicellulose and cellulose in the rumen [62,63,64]. In conclusion, administration of an appropriate dose of TV-PHFS can modulate the rumen microbiota of Kazakh lambs and improve the efficiency of fibrous material utilization. In this study, TV-PHFS supplementation significantly enriched fiber-degrading bacteria; however, rumen VFAs, growth performance, and serum metabolic indices showed no significant changes. Possible reasons include: the rumen microbiota exhibits functional redundancy, so changes in community structure may not alter fermentation end-product concentrations; measured VFA concentrations reflect the combined effects of production and absorption, and newly produced VFAs may be rapidly absorbed or pass out of the rumen; experimental duration, dosage, and physiological stage may also mask potential effects at the microbial level. Therefore, the observed microbial changes may mainly reflect improved fiber-degrading capacity; whether such microbial-level changes can ultimately translate into improved production performance warrants further investigation.

Meat quality serves as a crucial indicator for evaluating the edibility and consumer satisfaction associated with meat products. Crude fat content is recognized as a significant factor influencing muscle quality [65]. Previous studies indicated that in ruminant diets, substituting unconventional feeds for grains or directly supplementing tannin-based additives could improve meat quality without altering animal growth performance, by modifying the fatty acid profile and reducing fat deposition [66,67]. Smith [68] pointed out that intramuscular fat synthesis primarily utilized glucose as a carbon source. Supplying glucose or its precursors during early growth could promote intramuscular fat deposition. Huang et al. [69] demonstrated that dietary supplementation with 0.5% *Pseudostellaria heterophylla* extract (at a concentration of 40 g/L) in the feed of large yellow croaker markedly increased the levels of crude fat, crude protein, total amino acids, and n-3 polyunsaturated fatty acids within the muscle tissue, concurrently elevating the muscle EPA + DHA content. The current study observed that, in comparison to the control group, dietary supplementation with 0.3 kg/d of TV-PHFS significantly increased the crude fat content and 24 h L* value in the *longissimus dorsi* muscle of Kazakh lambs. This might be because TV-PHFS induced remodeling of the rumen microbial community, in which the enriched *Prevotella* might promote the degradation of carbohydrates in the rumen, thereby increasing the supply of gluconeogenic precursors and consequently promoting fat deposition in muscle [70]; meanwhile, changes in the composition and availability of microbial metabolites might also indirect effects on lipid metabolism in host muscle tissue. However, given that rumen fermentation parameters (including volatile fatty acids, pH, and ammonia nitrogen) did not show significant changes, the functional relationship between the enrichment of Prevotella and the observed increase in intramuscular fat deposition remained to be established. The elevation in intramuscular fat content contributed to increased light reflectance, improving the overall L* of the lamb meat [71]; additionally, the heightened intramuscular fat content enhanced flavor, tenderness, and juiciness attributes of the lamb meat [72]. Nonetheless, the electronic nose analysis results of this study indicated that dietary supplementation with 0.3 kg/d TV-PHFS did not significantly influence the volatile flavor compounds in lamb meat, potentially due to the limited sensitivity of existing electronic nose sensors to characteristic flavor compounds in lamb. Furthermore, the study found that supplementing the diet with 0.2 kg/d TV-PHFS significantly increased the b* value of the *longissimus dorsi* muscle after 45 min postmortem in Kazakh lambs. This might be related to the possibility that the bioactive substances in TV-PHFS could promote the oxidation of myoglobin and fat in muscle, and excessive lipid oxidation might lead to an increase in meat b* [73]. Additionally, in Kazakh lambs receiving 0.1 kg/d TV-PHFS, the content of hydrogen-like compounds in raw *longissimus dorsi* muscle was elevated, with alkanes demonstrating an increasing trend. This might be related to the possibility that TV-PHFS altered the fatty acid composition of muscle in Kazakh lambs, and the increased content of unsaturated fatty acids could accelerate fat oxidation [74], thereby potentially leading to an increase in aldehyde and alkane volatile compounds in raw meat. Although improvements in meat color and intramuscular fat content were observed, these were among the few statistically significant findings, whereas most other variables remained unaffected. This imbalance suggested that the effects of TV-PHFS might be limited to specific tissues or pathways rather than representing a broad metabolic response; the specific reasons remained to be further investigated.

It is noteworthy that the optimal dosage varied across parameters in this study: favorable changes in serum AST and total cholesterol appeared at 0.1 kg/d; alterations in b* at 45 min postmortem and the abundance of *Prevotellaceae_UCG-003* occurred at 0.2 kg/d; whereas significant changes in intramuscular fat content, the L* value of the *longissimus dorsi* at 24 h, and the relative abundances of *Treponema* and *Veillonellaceae_UCG_001* were detected only at 0.3 kg/d. This non-monotonic dose–response spectrum indicates that the multiple bioactive components in TV-PHFS may exert their biological effects through independent mechanisms and at distinct dose thresholds, rather than following a simple additive pattern of “the higher the dose, the stronger the effect”. This study had several limitations. The primary objective was to evaluate the overall application effects of TV-PHFS as a complete fermented product in the diet of Kazakh lambs, and the study was designed as a preliminary investigation simulating the direct supplementation approach commonly used in production practice. Therefore, isoenergetic and isonitrogenous diets were not formulated; however, the results might serve as dose references for future confirmatory studies using isonitrogenous and isoenergetic designs. The experiment prioritized different inclusion levels and did not include an unfermented *Pseudostellaria heterophylla* control group; therefore, the effects of TV-PHFS could not be strictly separated into those derived from the bioactive components of the medicinal plant, the fermentation process by *Trametes versicolor*, or fungal metabolites. Nutrient digestibility was not measured, and actual nutrient intake per lamb was not recorded, which limited the evaluation of the direct contribution of TV-PHFS to feed digestion and utilization efficiency. Functional prediction of the rumen microbiota was not performed; current inferences were based only on changes in microbial community structure, and specific metabolic pathway alterations could not be identified. We suggest that future studies should include an unfermented control, conduct digestion and metabolism trials, improve the characterization of bioactive components, and combine metagenomic or metabolomic approaches to more comprehensively elucidate the mechanisms of TV-PHFS.

## 5. Conclusions

Dietary supplementation with 0.3 kg/d of the TV-PHFS modulated the rumen fiber-degrading microbiota, promoted intramuscular fat deposition, and improved meat color, thereby enhancing lamb meat quality. However, no significant effects were observed on growth performance, antioxidant capacity, or immune status. Future studies will further investigate the metabolic pathways of the active components (such as polysaccharides and cyclic peptides) in TV-PHFS within the rumen of mutton lambs and the molecular mechanisms underlying their regulation of meat quality.

## Figures and Tables

**Table 1:** Composition and Nutritional Profile of the Basal Diet (Based on Dry Matter).

Feed Composition	Content/%
Maize stalks	42.51
Cottonseed hulls	42.51
Concentrate	14.98
Total	100
Nutritional Levels	
Energy (MJ/kg)	20.08
Crude ash	7.78
Crude protein	7.51
Crude fat	2.37
Neutral detergent fibre	55.18
Acid detergent fibre	41.32
Calcium	0.39
Phosphorus	0.35

Note: Each kg of concentrate contained: VA 8000 IU; VD_3_ 2400 IU; VE 20 IU; Cu 21 mg; Fe 78 mg; I 0.80 mg; Se 0.50 mg; Mn 80 mg; Zn 86 mg; Co 0.35 mg. The nutrient composition of the basal diet was based on calculated values.

**Table 2:** Effects of TV-PHFS on weight fluctuations in Kazakh lambs.

Items	Control Group	TV-PHFS Inclusion Levels (kg/d)			SEM	*p*-Value		
		0.1	0.2	0.3		Overall	Linear	Quadratic
Initial weight (kg)	32.1	32.6	32.8	32.6	1.170	0.997	0.871	0.895
Final weight (kg)	56.5	56.8	56.4	55.7	2.391	0.980	0.779	0.349
Average daily weight gain (g/d)	170	173	168	177	14.896	0.980	0.812	0.877
Carcass weight (kg)	28.4	29.6	26.6	26.3	0.526	0.081	0.044	0.431

TV-PHFS: *Trametes versicolor*-*Pseudostellaria heterophylla* fermented substrate. SEM: standard error of the mean. Control group: basal diet without TV-PHFS supplementation; 0.1: group supplemented with 0.1 kg/d of TV-PHFS; 0.2: group supplemented with 0.2 kg/d of TV-PHFS; 0.3: group supplemented with 0.3 kg/d of TV-PHFS.

**Table 3:** Effects of TV-PHFS on biochemical indicators in Kazakh lamb blood.

Items	Control Group	TV-PHFS Inclusion Levels (kg/d)			SEM	*p*-Value		
		0.1	0.2	0.3		Overall	Linear	Quadratic
TP (g/L)	64.20	63.40	64.50	65.90	0.749	0.753	0.421	0.520
ALB (g/L)	35.00	34.13	35.17	33.97	0.332	0.537	0.518	0.814
GLB (g/L)	29.20	29.27	29.33	31.93	0.713	0.510	0.241	0.410
ALB/GLB	1.20	1.17	1.20	1.13	0.032	0.878	0.591	0.754
ALT (U/L)	24.33	19.33	27.00	25.67	1.276	0.147	0.266	0.425
AST (U/L)	144.00	115.00	135.33	141.67	4.499	0.059	0.672	0.032
ALT/AST	0.17	0.17	0.20	0.18	0.008	0.615	0.475	0.510
BUN (mmol/L)	2.72	2.46	2.73	4.49	0.357	0.151	0.077	0.138
GLU (mmol/L)	4.26	4.50	3.71	3.86	0.245	0.712	0.425	0.939
ALP (U/L)	440.67	457.00	392.00	518.00	39.026	0.779	0.675	0.541
TG (mmol/L)	0.23	0.32	0.25	0.23	0.021	0.479	0.815	0.252
TC (mmol/L)	1.47	2.03	1.62	1.47	0.095	0.096	0.561	0.047
HDL-C (mmol/L)	0.88	1.15	0.88	0.84	0.053	0.123	0.341	0.120
LDL-C (mmol/L)	0.41	0.62	0.56	0.44	0.043	0.295	0.900	0.078

TP: Total protein; ALB: Albumin; GLB: Globulin; ALB/GLB: Albumin/globulin ratio; ALT: Alanine aminotransferase; AST: Aspartate aminotransferase; ALT/AST: Alanine aminotransferase/aspartate aminotransferase ratio; BUN: Urea nitrogen; GLU: Glucose; ALP: Alkaline phosphatase; TG: Triglycerides; TC: Total cholesterol; HDL-C: High-density lipoprotein cholesterol; LDL-C: Low-density lipoprotein cholesterol. TV-PHFS: *Trametes versicolor*-*Pseudostellaria heterophylla* fermented substrate. SEM: standard error of the mean. Control group: basal diet without TV-PHFS supplementation; 0.1: group supplemented with 0.1 kg/d of TV-PHFS; 0.2: group supplemented with 0.2 kg/d of TV-PHFS; 0.3: group supplemented with 0.3 kg/d of TV-PHFS.

**Table 4:** Effects of TV-PHFS on antioxidant indicators in Kazakh lamb blood.

Items	Control Group	TV-PHFS Inclusion Levels (kg/d)			SEM	*p*-Value		
		0.1	0.2	0.3		Overall	Linear	Quadratic
SOD (U/mL)	141.57	144.56	169.89	152.34	4.642	0.110	0.129	0.215
CAT (U/mL)	0.60	0.41	0.66	0.72	0.063	0.363	0.281	0.349
GSH-Px (U/mL)	100.22	93.0	78.20	102.92	4.713	0.262	0.867	0.105
MDA (nmol/mL)	3.19	2.56	1.79	2.08	0.251	0.220	0.077	0.340
T-AOC (mmol/L)	0.99	0.93	1.06	1.05	0.026	0.219	0.163	0.586

SOD: Superoxide dismutase; CAT: Catalase; GSH-Px: Glutathione peroxidase; MDA: Malondialdehyde; T-AOC: Total antioxidant capacity. TV-PHFS: *Trametes versicolor*-*Pseudostellaria heterophylla* fermented substrate. SEM: standard error of the mean. Control group: basal diet without TV-PHFS supplementation; 0.1: group supplemented with 0.1 kg/d of TV-PHFS; 0.2: group supplemented with 0.2 kg/d of TV-PHFS; 0.3: group supplemented with 0.3 kg/d of TV-PHFS.

**Table 5:** Effect of TV-PHFS on immune indicators in Kazakh lamb blood.

Items	Control Group	TV-PHFS Inclusion Levels (kg/d)			SEM	*p*-Value		
		0.1	0.2	0.3		Overall	Linear	Quadratic
IgG (mg/mL)	3.53	4.83	3.63	6.01	0.776	0.700	0.428	0.756
IgM (ug/mL)	144.90	132.25	135.40	174.56	11.109	0.583	0.401	0.297
IgA (ug/mL)	285.56	352.28	273.29	240.23	25.473	0.524	0.385	0.368
IL-1β (pg/mL)	46.20	37.90	62.63	50.63	5.614	0.529	0.483	0.877
TNF-α (pg/mL)	80.46	88.30	130.64	111.98	13.560	0.610	0.315	0.655
IL-10 (pg/mL)	111.11	194.41	188.04	208.78	27.771	0.664	0.312	0.612

IgG: Immunoglobulin G; IgM: Immunoglobulin M; IgA: Immunoglobulin A; IL-1β: Interleukin-1 beta; TNF-α: Tumor necrosis factor alpha; IL-10: Interleukin-10. TV-PHFS: *Trametes versicolor*-*Pseudostellaria heterophylla* fermented substrate. SEM: standard error of the mean. Control group: basal diet without TV-PHFS supplementation; 0.1: group supplemented with 0.1 kg/d of TV-PHFS; 0.2: group supplemented with 0.2 kg/d of TV-PHFS; 0.3: group supplemented with 0.3 kg/d of TV-PHFS.

**Table 6:** Changes in rumen fermentation in Kazakh lambs fed different quantities of TV-PHFS.

Items	Control GROUP	TV-PHFS Inclusion Levels (kg/d)			SEM	*p*-Value		
		0.1	0.2	0.3		Overall	Linear	Quadratic
pH	5.71	5.67	5.32	5.76	0.120	0.621	0.868	0.377
Acetic acid (mmol/L)	72.30	79.41	89.48	65.89	6.639	0.692	0.890	0.314
Propionic acid (mmol/L)	11.88	13.57	16.22	13.54	0.936	0.485	0.395	0.283
Acetate-to-propionate ratio	5.96	5.82	5.53	4.74	0.246	0.323	0.096	0.507
Isobutyric acid (mmol/L)	1.00	1.15	1.43	0.94	0.099	0.336	0.926	0.133
Butyric acid (mmol/L)	7.49	11.98	11.66	9.52	0.810	0.165	0.385	0.046
Isovaleric acid (mmol/L)	0.96	1.15	1.28	0.82	0.143	0.738	0.839	0.328
Valeric acid (mmol/L)	0.55	0.64	0.86	0.64	0.064	0.403	0.412	0.256
TVFA (mmol/L)	94.18	107.89	120.93	91.35	8.362	0.639	0.956	0.257
NH_3_-N (mmol/L)	2.82	7.49	5.83	5.13	0.727	0.133	0.363	0.059

TVFA: Total volatile fatty acids; NH_3_-N: Ammonia nitrogen. TV-PHFS: *Trametes versicolor*-*Pseudostellaria heterophylla* fermented substrate. SEM: standard error of the mean. Control group: basal diet without TV-PHFS supplementation; 0.1: group supplemented with 0.1 kg/d of TV-PHFS; 0.2: group supplemented with 0.2 kg/d of TV-PHFS; 0.3: group supplemented with 0.3 kg/d of TV-PHFS.

**Table 7:** Effects of TV-PHFS on the meat quality of Kazakh lambs.

Items	Control Group	TV-PHFS Inclusion Levels (kg/d)			SEM	*p*-Value		
		0.1	0.2	0.3		Overall	Linear	Quadratic
pH 45 min	6.40	6.20	6.29	6.32	0.079	0.883	0.873	0.530
pH 24 h	5.53	5.62	5.68	5.62	0.034	0.475	0.279	0.291
L* 45 min	35.78	39.81	35.08	39.46	0.887	0.102	0.357	0.908
L* 24 h	40.32 ^b^	41.23 ^ab^	38.33 ^b^	43.80 ^a^	0.735	0.032	0.138	0.056
a* 45 min	27.29	24.51	25.53	24.52	0.792	0.628	0.360	0.613
a* 24 h	17.33	19.82	19.28	20.01	0.881	0.753	0.410	0.660
b* 45 min	1.51 ^b^	2.28 ^ab^	3.26 ^a^	1.44 ^b^	0.262	0.016	0.615	0.005
b* 24 h	7.63	6.42	6.43	7.58 ^a^	0.408	0.620	0.969	0.209
Backfat thickness (mm)	2.21	3.82	3.14	2.92	0.225	0.060	0.372	0.028
Shear force (N)	125.51	99.79	125.36	122.33	7.539	0.632	0.828	0.498

Means with different superscripts within the same row differ significantly (*p* < 0.05). L*: lightness; a*: redness; b*: yellowness. TV-PHFS: *Trametes versicolor*-*Pseudostellaria heterophylla* fermented substrate. SEM: standard error of the mean. Control group: basal diet without TV-PHFS supplementation; 0.1: group supplemented with 0.1 kg/d of TV-PHFS; 0.2: group supplemented with 0.2 kg/d of TV-PHFS; 0.3: group supplemented with 0.3 kg/d of TV-PHFS.

**Table 8:** Effects of TV-PHFS on the nutrient composition of the *longissimus dorsi* muscle in Kazakh lamb.

Items/%	Control Group	TV-PHFS Inclusion Levels (kg/d)			SEM	*p*-Value		
		0.1	0.2	0.3		Overall	Linear	Quadratic
Moisture (%)	74.00	74.38	74.90	73.50	0.297	0.412	0.721	0.148
Ash (%)	8.94	8.62	7.79	7.69	0.338	0.506	0.151	0.875
Protein (%)	75.72	74.28	76.09	73.11	0.748	0.497	0.383	0.616
Fat (%)	10.92 ^b^	13.97 ^ab^	11.81 ^b^	16.76 ^a^	0.829	0.047	0.029	0.525

Means with different superscripts within the same row differ significantly (*p* < 0.05). TV-PHFS: *Trametes versicolor*-*Pseudostellaria heterophylla* fermented substrate. SEM: standard error of the mean. Control group: basal diet without TV-PHFS supplementation; 0.1: group supplemented with 0.1 kg/d of TV-PHFS; 0.2: group supplemented with 0.2 kg/d of TV-PHFS; 0.3: group supplemented with 0.3 kg/d of TV-PHFS.

**Table 9:** Names of Electronic Nose Sensors and Their Corresponding Representative Sensitive Substances.

Probe Number	Matter
S1	Propane, Smoke
S2	Alcohol, Smoke, Isobutane, Formaldehyde
S3	Ozone
S4	Hydrogen Sulphide
S5	Ammonia
S6	Toluene, Acetone, Ethanol, Hydrogen
S7	Methane, Natural Gas, Biogas
S8	Liquefied Petroleum Gas
S9	Toluene, Formaldehyde, Benzene, Alcohol, Acetone
S10	Hydrogen
S11	Liquefied Petroleum Gas, Alkanes
S12	Liquefied Petroleum Gas, Methane
S13	Methane
S14	Flammable Gases, Smoke

## Data Availability

The raw data supporting the conclusions of this article will be made available by the authors on request.

## Abbreviations

The following abbreviations are used in this manuscript:

TV-PHFS	*Trametes versicolor*-*Pseudostellaria heterophylla* fermented substrate
TP	Total protein
ALB	Albumin
GLB	Globulin
BUN	Urea nitrogen
GLU	Glucose
ALT	Alanine aminotransferase
AST	Aspartate aminotransferase
TC	Total cholesterol
TG	Triglycerides
HDL-C	High-density lipoprotein cholesterol
LDL-C	Low-density lipoprotein cholesterol
T-AOC	Total antioxidant capacity
SOD	Superoxide dismutase
CAT	Catalase
GSH-Px	Glutathione peroxidase
MDA	Malondialdehyde
VFAs	Volatile fatty acids
TVFA	Total Volatile Fatty Acids
IgA	Immunoglobulin A
IgG	Immunoglobulin G
IgM	Immunoglobulin M
IL-1β	Interleukin-1β
IL-10	Interleukin-10
TNF-α	Tumor necrosis factor
NH_3_-N	Ammonia nitrogen
ASVs	AmpliconSequence Variants
EPA	Eicosapentaenoic acid (20:5n-3)
DHA	Docosahexaenoic acid (22:6n-3)
